# STING/Type I Interferon Pathway Activation Confers Multimodal Therapeutic Benefits in Osteomyelitis

**DOI:** 10.1002/advs.77892

**Published:** 2026-10-26

**Authors:** Yang Chen, Jianhua Lv, Yuanqing Cai, Xuhui Yuan, Hongxin Hu, Jiayu Li, Yiming Lin, Hongyan Li, Lan Lin, Huangfeng Lin, Chaofan Zhang, Qijin Wang, Ying Huang, Zida Huang, Wenbo Li, Susheng Niu, Jianhua Lin, Bin Yang, Xinyu Fang, Wenming Zhang

**Affiliations:** ^1^ Department of Orthopedic Surgery National Regional Medical Center Binhai Campus of the First Affiliated Hospital Fujian Medical University Fuzhou China; ^2^ Department of Orthopedic Surgery Affiliated Hospital of Putian University Putian China; ^3^ Department of Orthopedic Surgery The First Affiliated Hospital of Fujian Medical University Fuzhou China; ^4^ Fujian Provincial Institute of Orthopedics, the First Affiliated Hospital Fujian Medical University Fuzhou China; ^5^ Key Laboratory of Orthopedics & Traumatology of Traditional Chinese Medicine and Rehabilitation Ministry of Education Fujian University of Traditional Chinese Medicine Fuzhou China; ^6^ Department of Laboratory Medicine The First Affiliated Hospital of Fujian Medical University Fuzhou China

**Keywords:** abscess, bone destruction, osteomyelitis, pain, stimulator of interferon genes

## Abstract

Osteomyelitis is an inflammatory bone disease caused by pathogenic microorganisms and characterized by severe pain, extensive bone destruction, and reduced antibiotic efficacy due to local abscess formation. This study investigates the therapeutic potential of activating the STING (stimulator of interferon genes) pathway in osteomyelitis. In murine femoral osteomyelitis models induced by *Staphylococcus aureus* (*S. aureus*), STING agonists alleviate osteomyelitis‐associated pain, suppress infection‐induced bone destruction, and reduce local abscess formation. These effects depend on host STING/type I interferon (IFN‐I) signaling and involve three mechanisms: inhibition of nociceptor activation by pathogens, suppression of osteoclast differentiation and function without affecting osteoblast activity, and downregulation of interleukin‐1β (IL‐1β), leading to reduced neutrophil recruitment and abscess formation. By reducing abscess formation as a physical barrier, STING agonists enhance the antibacterial efficacy of conventional antibiotics such as vancomycin at the site of infection. In summary, these findings suggest that the synergistic mechanisms activated by STING represent a promising therapeutic target for alleviating pain and improving clinical outcomes in osteomyelitis.

## Graphical abstract

STING/type I interferon signaling counteracts osteomyelitis through coordinated control of pain, bone destruction, and abscess formation. It inhibits pathogen‐induced nociceptor activation, restrains osteoclast‐mediated bone resorption, and suppresses IL‐1β‐driven neutrophil recruitment. Reduced abscess formation further facilitates antibiotic penetration and enhances the antibacterial efficacy of conventional antibiotics, supporting STING activation as a multimodal therapeutic strategy.

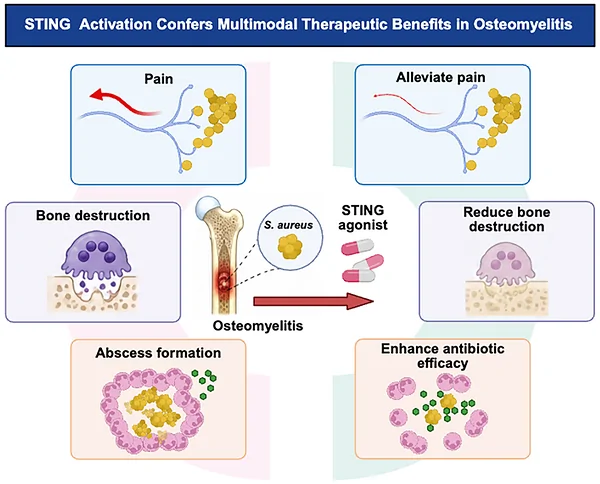

## 1 Introduction

Osteomyelitis is an inflammatory bone disease caused by pathogenic microorganisms, characterized by intense pain and severe bone destruction [1, 2]. Antibiotics constitute a primary therapeutic approach [2, 3]; however, clinical evidence indicates a 20%–30% treatment failure rate in osteomyelitis management, with poorly controlled infections potentially leading to disability or life‐threatening complications [4, 5, 6, 7]. Treatment failure often necessitates prolonged antibiotic courses, which increases risks of antibiotic‐related adverse events [8, 9], and may further compromise systemic immune function through gut microbiota dysbiosis, establishing a vicious cycle [10].

Staphylococcus aureus (S. aureus) is implicated in over 50% of osteomyelitis cases [11]. Pathogenic bacteria directly activate intraosseous and periosteal nociceptors by releasing virulence factors, eliciting pain [12]. Concurrently, they disrupt the balance between osteogenesis and osteoclastogenesis [13]. For instance, S. aureus promotes osteoclast differentiation while suppressing osteoblast function through multiple mechanisms—such as secreting virulence factor Staphylococcal Protein A (SpA)—ultimately driving pathological bone destruction in osteomyelitis [14, 15, 16]. Additionally, bacteria evade antibiotic killing by persisting within bone lacunae, osteocytes, biofilms, or abscesses [11, 17, 18, 19]. This evasion capability underscores the need for novel strategies to enhance antibacterial efficacy against osteomyelitic pathogens.

Osteomyelitis and osseous metastatic tumors share key clinicopathological characteristics: both manifest with pain and pathological bone destruction [20, 21]. Recently, stimulator of interferon genes (STING) pathway agonists have shown significant promise in cancer therapy. For instance, studies of bone metastases demonstrate that STING agonists (e.g., DMXAA) exert multimodal effects via activation of the cGAS‐STING–IFN‐I (type I interferon) axis: First, STING‐induced IFN‐I activates dendritic cells and cytotoxic T lymphocytes, enhancing antitumor immunity; second, STING signaling suppresses tumor‐mediated osteoclast differentiation, reducing osteolytic destruction; moreover, STING agonists exhibit intrinsic analgesic properties [22]. These findings provide novel therapeutic perspectives for osteomyelitis management. Previous studies confirm STING pathway activation influences immune cell function during bacterial infections through distinct mechanisms [23, 24], suggesting that exogenous STING agonists may regulate endogenous immune signaling to counteract pathogen immune evasion in bone infections.

This study systematically evaluates the role of the STING pathway in osteomyelitis pathogenesis and explores its therapeutic potential. We established osteomyelitis models utilizing multiple genetically modified murine strains and administered STING agonists as intervention. Our findings conclusively demonstrate the significant effects of STING agonists in alleviating osteomyelitic pain, mitigating bone destruction, and enhancing the antibacterial efficacy of antibiotics.

## 2 Results

### 2.1 STING Agonists Alleviate Osteomyelitis‐Related Pain

*Staphylococcus aureus* is the most common pathogen in clinical cases of osteomyelitis, particularly methicillin‐resistant *Staphylococcus aureus* (MRSA), which often leads to persistent osteomyelitis [11]. We injected MRSA strains (USA300, 2 µl of 1 × 10^6^ CFU) into the femurs of C57BL/6 mice to establish a mouse model of osteomyelitis. To observe whether the STING agonist could exert analgesic effects in the osteomyelitis mouse model, we administered vehicle or DMXAA (20 mg/kg) via intraperitoneal injection on days 3 and 7 post‐infection. We assessed the differences in mechanical and thermal hyperalgesia between the two groups of mice multiple times from 1 to 14 days post‐infection using the Von Frey test and the Hargreaves test (Figure 1A). We observed that the STING agonist (DMXAA) significantly alleviated pain in mice during the course of osteomyelitis (Figure 1B–D). There were no significant differences in body weight changes or liver function (ALT, AST) and kidney function (Cr, BUN) indicators between the two groups during the treatment period (Figure 1E–I), indicating that the drug is relatively safe. Given that Streptococcus is one of the common pathogens in osteomyelitis, second only to *Staphylococcus aureus* [25], we also tested the analgesic effect of DMXAA in a Streptococcus‐induced osteomyelitis mouse model and found that DMXAA had a significant analgesic effect (Figure S1A–C), suggesting that the analgesic effect of DMXAA in osteomyelitis is not specific to a particular strain but represents a broader effect.

**FIGURE 1:**
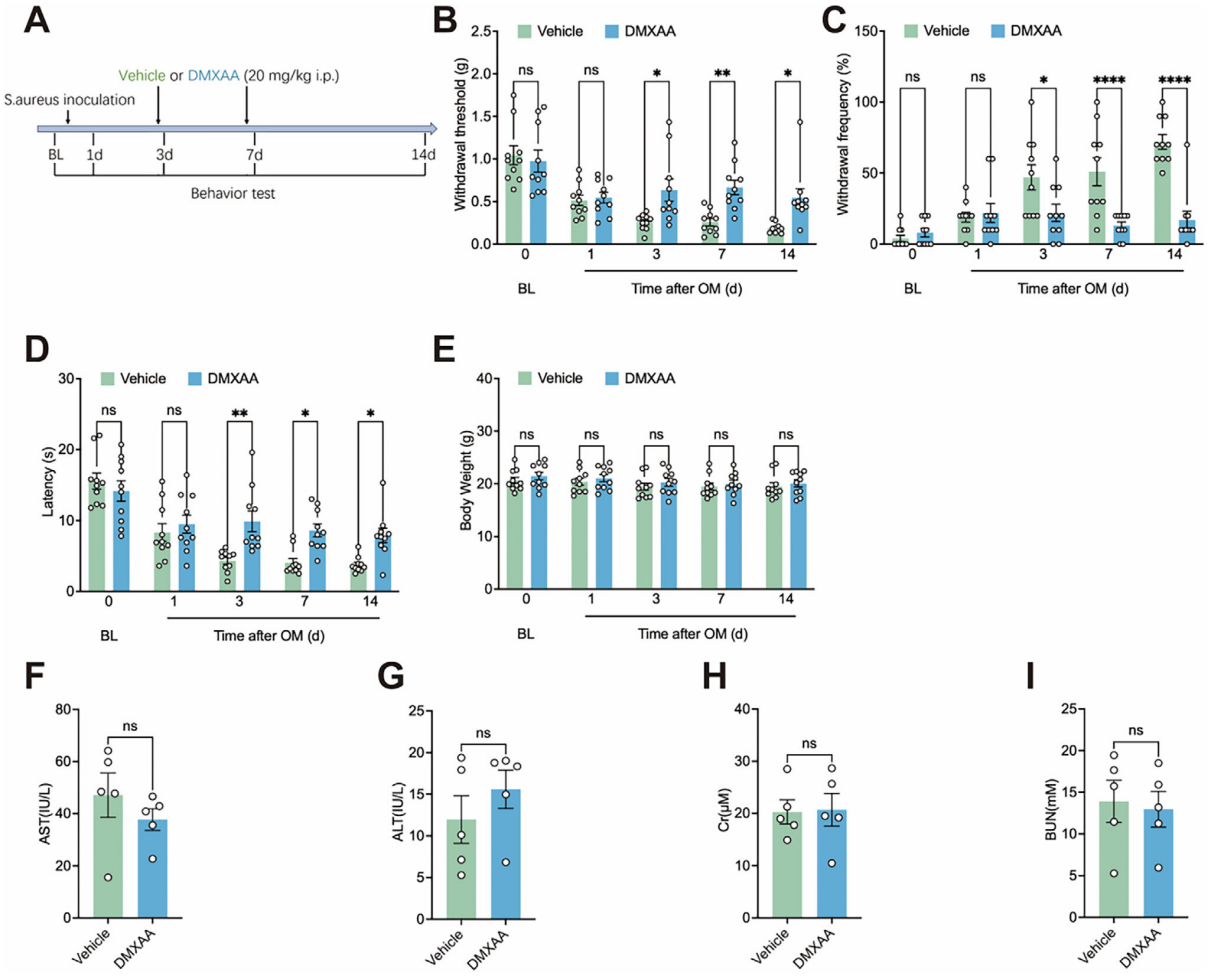
STING agonists alleviates pain in a murine model of *S. aureus*‐induced osteomyelitis. (A) Schematic diagram of intraperitoneal injection of vehicle or DMXAA (20 mg/kg) in WT mice on day 3 and day 7 after *S. aureus* (2µL, 1 × 10^6^CFU) inoculation, followed by behavior tests within 14 days post‐infection; (B–D) Development of mechanical (B, C) and thermal (D) hyperalgesia over time in mice with *S. aureus*‐induced osteomyelitis treated with vehicle or DMXAA (n = 10 mice/group). Mechanical hyperalgesia was gauged by the paw withdrawal threshold (B) and the response frequency to a 0.16 g von Frey filamentthe withdrawal frequency (C). Thermal hyperalgesia (D) was assessed using the Hargreaves test. The time point labeled “0” signifies the baseline condition prior to the onset of infection; (E) Temporal changes in body weight after osteomyelitis induction in vehicle‐ or DMXAA‐treated mice. The time point labeled ‘0’ signifies the baseline condition prior to the onset of infection. n = 10 mice/group; (F–I) The assessment of hepatotoxicity and nephrotoxicity through the analysis of serum levels of AST (F), ALT (G), Cr (H), and BUN (I) on the 14th day after OM in vehicle‐ or DMXAA‐treated mice. n = 5 mice/group. BL: Baseline. OM: Osteomyelitis. Statistical analysis: (B–E) Two‐way ANOVA, Bonferroni's post‐hoc tests. (F–I) Student's *t*‐tests. ^*^ *P*<0.05, ^**^ *P*<0.01, ^***^ *P*<0.001, ^****^ *P*<0.0001. ns = not significant. All data are presented as mean ± SEM.

To clarify whether the effects observed are unique to DMXAA or applicable to other STING agonists, we repeated the experiments using ADU‐S100, which has been reported to be effective in humans [26]. Our results indicated that ADU‐S100 also alleviated pain associated with osteomyelitis (Figure S1D–F). This suggests that multiple STING agonists may exhibit similar analgesic effects and that such effects may not be limited to mice alone.

### 2.2 STING Agonists Alleviate Osteomyelitis‐Related Bone Destruction

Osteomyelitis is often associated with significant destruction of bone tissue, leading to a marked reduction in the structural integrity of the bone, which can subsequently result in pathological fractures and limb deformities [27, 28]. Previous studies have indicated that bacterial toxins can promote osteoclast differentiation, resulting in bone destruction during osteomyelitis [15]. To evaluate the impact of STING agonists on bone destruction associated with osteomyelitis, we dynamically assessed the extent of bone destruction in vehicle‐ and DMXAA‐treated groups using x‐ray imaging at various time points post‐infection. Bone destruction scoring was conducted based on x‐ray images, as reported in the literature by Norden [29]. We observed that DMXAA treatment significantly alleviated bone destruction compared to the vehicle group at days 10 and 20 post‐infection with *S. aureus* (Figure 2A,B).

**FIGURE 2:**
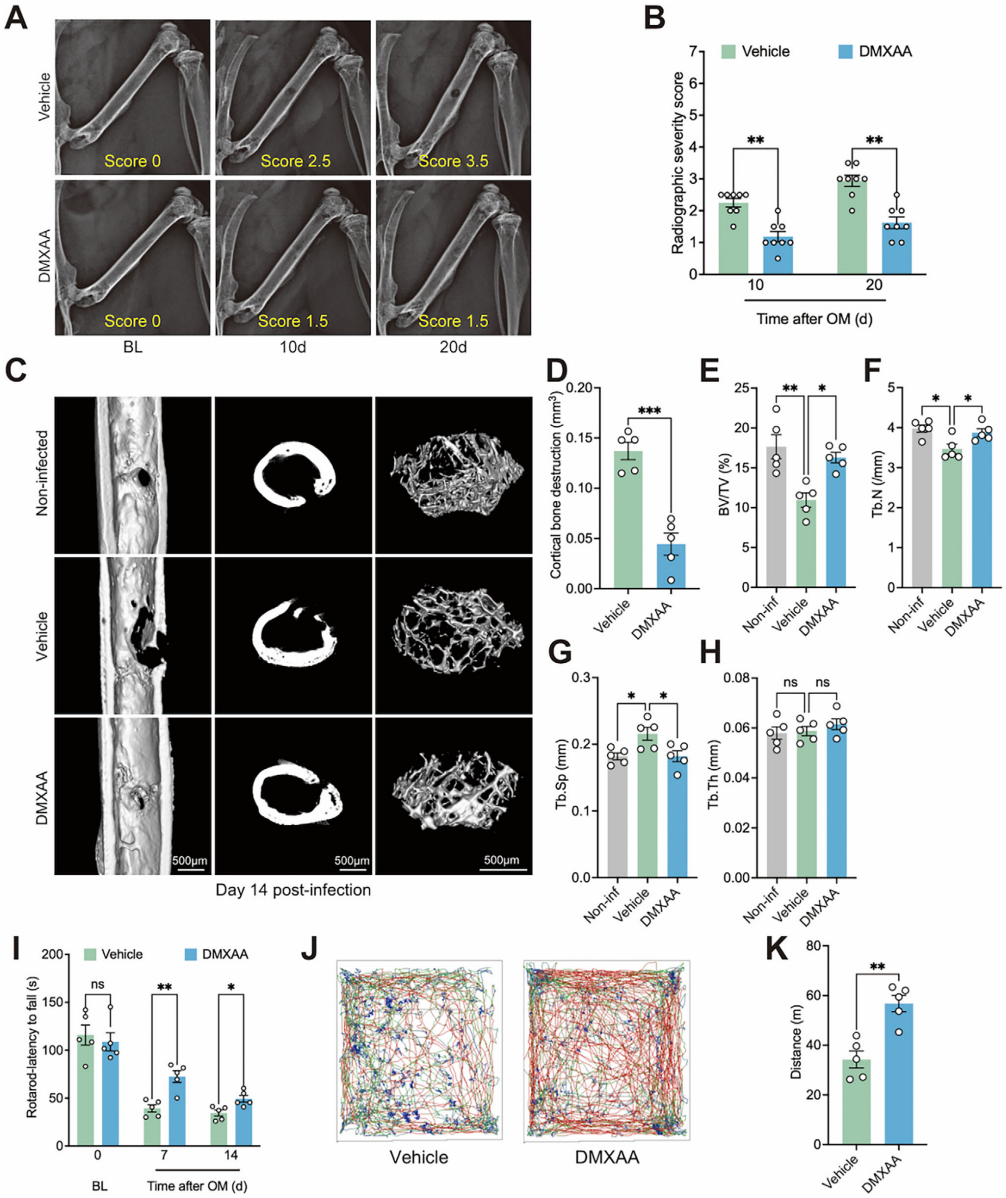
STING agonist alleviates *S. aureus*‐induced bone destruction in osteomyelitis. (A, B) Typical x‐ray images of femur (A) and radiographic severity scores (B) assess bone destruction in *S. aureus*‐induced osteomyelitis at days 10 and 20 post‐infection in vehicle‐ or DMXAA‐treated mice. n = 8 mice/group; (C–H) Micro‐CT analysis of femurs at day 14 post‐infection. (C) Representative micro‐CT images of femurs from non‐infected mice and vehicle‐ or DMXAA‐treated mice with S. aureus‐induced osteomyelitis (scale bars: 500 µm). (D) Quantification of cortical bone destruction in vehicle‐ or DMXAA‐treated mice with S. aureus‐induced osteomyelitis. (E–H) Quantitative analysis of bone microstructure in vehicle‐ or DMXAA‐treated mice with *S. aureus*‐induced osteomyelitis, compared with non‐infected mice. n = 5 mice/group; (I–K) Comparison of motor function between vehicle‐ and DMXAA‐treated mice with osteomyelitis, including latency to fall (s) in the rotarod test at baseline, day 7, and day 14 post‐infection (I), representative open field traces from a 15‐min test (J), and quantification of total distance traveled (K) at day 14 post‐infection. The time point labeled “0” signifies the baseline condition prior to infection. n = 5 mice/group. BV/TV: Bone Volume/Total Volume. Non‐inf: Non‐infected. Tb.N: Trabecular number. Tb.Sp: Trabecular separation. Tb.Th: Trabecular thickness. BL: Baseline. OM: Osteomyelitis. Statistical analysis: (B, I) Two‐way ANOVA, Bonferroni's post‐hoc tests. (E‐H) One‐way ANOVA, Tukey's post‐hoc tests. (D, K) Student's *t*‐tests. ^*^ *P*<0.05, ^**^ *P*<0.01, ^***^ *P*<0.001. ns = not significant. All data are presented as mean ± SEM.

To further characterize the structural changes in the femur following osteomyelitis, we performed micro‐CT analysis of the infected femurs at day 14 post‐infection. Compared with the vehicle‐treated group, DMXAA‐treated mice exhibited significantly less cortical bone destruction two weeks after *S. aureus* infection (Figure 2C,D). Consistent with these findings, quantitative analysis revealed that DMXAA treatment significantly increased the bone volume fraction (BV/TV) and trabecular number (Tb.N), while decreasing trabecular separation (Tb.Sp), compared with vehicle treatment (Figure 2E–H). Collectively, these findings indicate that activation of STING with DMXAA markedly attenuates *S. aureus*‐induced bone destruction in the femur. To determine whether this bone‐protective effect was also observed with another STING agonist, we repeated the experiments using ADU‐S100 and found that ADU‐S100 similarly alleviated bone destruction in *S. aureus*‐induced osteomyelitis (Figure S2A,B).

In clinical practice, pain and bone destruction limit patient mobility and activity willingness, thereby diminishing quality of life [30]. Consequently, addressing this functional decline represents an important clinical priority. To determine whether STING activation by DMXAA improves motor function in mice with osteomyelitis, motor performance was assessed at different time points post‐infection using the rotarod test and open field test. DMXAA treatment significantly improved motor performance, as evidenced by increased latency to fall in the rotarod test and greater total distance traveled in the open field test (Figure 2I–K). Further experiments revealed that ADU‐S100 also improved motor function in osteomyelitis mice (Figure S2C–E). Collectively, our results suggest that the STING agonist may contribute to the recovery of motor function in osteomyelitis by mitigating pain and halting bone destruction.

### 2.3 STING Agonists Alleviate Osteomyelitis‐Related Pain and Bone Destruction via the STING/IFN‐I Signaling Pathway

To validate that the analgesic and bone‐protective effects of the STING agonist in osteomyelitis are STING‐mediated, we constructed osteomyelitis models using wild‐type (WT) mice and STING gene knockout mice (STING^gt/gt^). On days 3 and 7 post‐infection, we administered DMXAA (20 mg/kg) via intraperitoneal injection and measured the variations in nociceptive thresholds in each group of mice before and after infection. We found that the STING^gt/gt^ mice exhibited significantly lower mechanical and thermal pain thresholds compared to the WT mice prior to infection. Furthermore, after the intraperitoneal injection of DMXAA, the STING^gt/gt^ mice did not experience pain relief (Figure S3A–C), indicating that the analgesic effect of DMXAA during the osteomyelitis process is mediated by STING. Additionally, we evaluated the extent of bone destruction in different groups of osteomyelitis mice treated with DMXAA using x‐ray imaging. Compared with the WT group, DMXAA treatment did not improve the bone destruction scores in the STING^gt/gt^ mice on days 10 and 20 post‐infection (Figure S3D,E). We noted that there were no differences in body weight changes among the different groups of mice over the 2‐week duration of the in vivo experiments (Figure S3F). Therefore, the above experimental results indicate that the protective effects of DMXAA against pain and bone destruction caused by osteomyelitis are mediated by STING.

The release of type I interferons (including IFN‐α and IFN‐β) is regulated by STING [31]. It remains unclear whether type I interferon (IFN‐I) contributes significantly to the mechanisms by which STING agonists alleviate pain and bone destruction in osteomyelitis. We analyzed the expression levels of IFN‐α and IFN‐β in serum and bone tissue using ELISA. The results showed that 4 h after DMXAA treatment, the levels of IFN‐α and IFN‐β in both serum and bone were significantly increased, with this elevation still observable 24 h post‐administration (Figure 3A–D), confirming that the STING agonist DMXAA indeed promotes substantial expression both systemically and locally within bone tissue. To further investigate the role of IFN‐I in the action of the STING agonist, we bred *Ifnar1* gene knockout mice (*Ifnar1^−/−^*), which possess a defect in one of the IFN‐I receptor subunits, rendering their IFN‐I receptors non‐functional. We infected the femurs of *Ifnar1^−/−^* mice and their littermate control mice (*Ifnar1^+/+^*) with *S. aureus* to establish an osteomyelitis model. Similarly, on days 3 and 7 post‐infection, we administered DMXAA or vehicle via intraperitoneal injection and assessed the variations in nociceptive thresholds among the groups before and after infection. The results indicated that the *Ifnar1^−/−^* group exhibited significantly lower mechanical and thermal pain thresholds compared to the control group prior to infection, and after intraperitoneal injection of DMXAA, the *Ifnar1^−/−^* mice did not experience pain relief (Figure 3E–G). On the other hand, x‐ray imaging scores revealed that the *Ifnar1^−/−^* group did not experience a reduction in bone destruction following DMXAA treatment, whereas the littermate controls showed significant reduced bone destruction post‐treatment (Figure 3H,I). No differences in body weight were observed among the groups (Figure 3J). These experimental results demonstrate that the analgesic and bone‐protective effects of the STING agonist DMXAA during the course of osteomyelitis are highly dependent on IFN‐I and its receptors.

**FIGURE 3:**
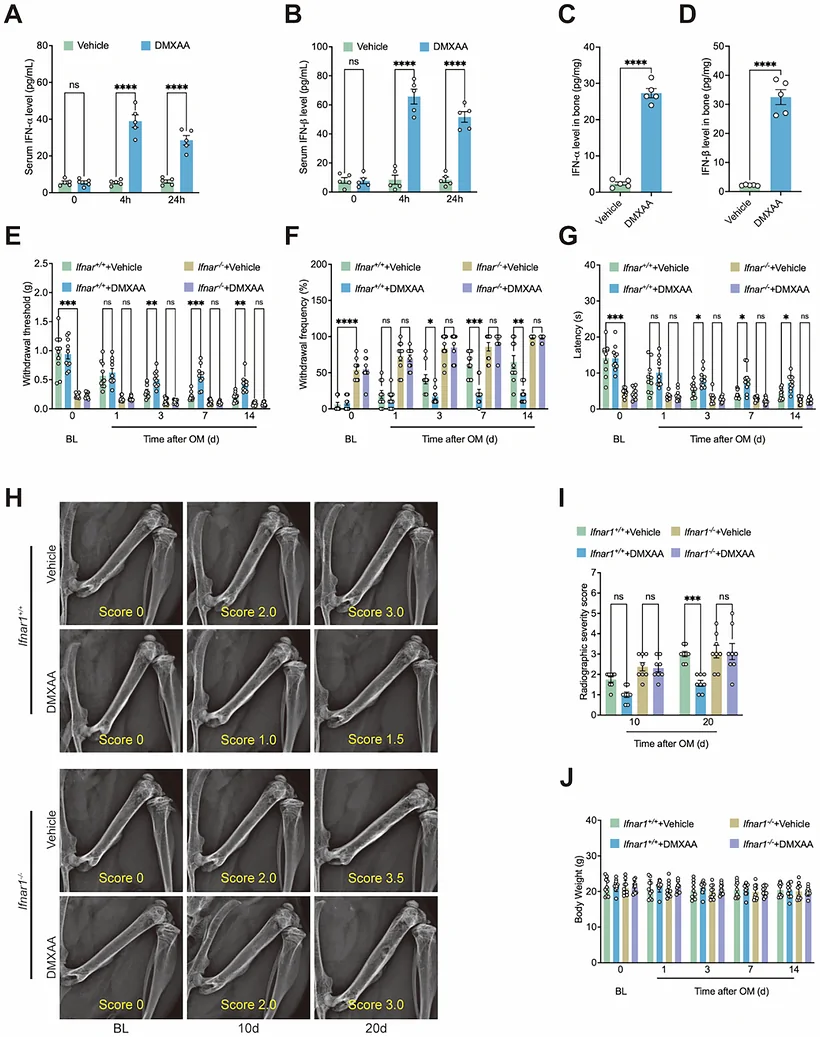
The analgesic and bone‐protective effects of STING agonist DMXAA are dependent on the IFN‐I signaling pathway. (A–D) ELISA analysis of IFN‐α and IFN‐β levels in serum (A, B) and bone (C, D) of mice with *S. aureus*‐induced osteomyelitis. Serum was collected before (0 h) and after (4, 24 h) vehicle or DMXAA treatment on day 3 post‐infection. Bone tissue was harvested at the 4 h post‐treatment time point. n = 5 mice/group; (E–G) Temporal changes in mechanical (E, F) and thermal (G) hyperalgesia in vehicle‐ or DMXAA‐treated *Ifnar1^+/+^* and *Ifnar1^−/−^* mice with *S. aureus*‐induced osteomyelitis. n = 10 mice/group; (H, I) Typical x‐ray images of femur (H) and radiographic severity scores (I) assess bone destruction in *S.aureus*‐induced osteomyelitis at days 10 and 20 post‐infection in *Ifnar1^+/+^* and *Ifnar1^−/−^* mice treated with either vehicle or DMXAA. n = 8 mice/group; J) Temporal changes in body weight after osteomyelitis induction in vehicle‐ or DMXAA‐treated *Ifnar1^+/+^* and *Ifnar1^−/−^* mice. n = 10 mice/group. BL: Baseline. OM: Osteomyelitis. Statistical analysis: (A, B, E, F, G, I, J) Two‐way ANOVA, Bonferroni's post‐hoc tests. (C, D) Student's *t*‐tests. ^*^ *P*<0.05, ^**^ *P*<0.01, ^***^ *P*<0.001, ^****^ *P*<0.0001. ns = not significant. All data are presented as mean ± SEM.

### 2.4 STING Agonists Inhibit *Staphylococcus aureus*‐Induced Activation of Nociceptive Neurons via the STING/IFN‐I Signaling Pathway

DMXAA administration may inhibit bacterial osteomyelitis‐induced bone pain through a combination of mechanisms, including reducing bacterial load, decreasing inflammatory factors, minimizing bone destruction, or directly inhibiting nociceptors. Previous literature has reported that *S. aureus* can directly activate nociceptive neurons to produce pain [12]. We hypothesized that STING agonists might achieve analgesic effects by inhibiting the activation of nociceptive neurons induced by *S. aureus*. To test this hypothesis, we isolated and cultured L3‐L5 dorsal root ganglion (DRG) neurons and treated them with DMXAA (50 µm) for 4 h. Following this, we added supernatant from *S. aureus* cultures and utilized calcium imaging techniques to detect the activation of DRG neurons in the different treatment groups. Our results indicated that compared to the vehicle group, there were significantly fewer DRG neurons activated in the DMXAA group (Figure 4A,B). This indicates that the STING agonist DMXAA can inhibit the direct activation of DRG neurons by *S. aureus*.

**FIGURE 4:**
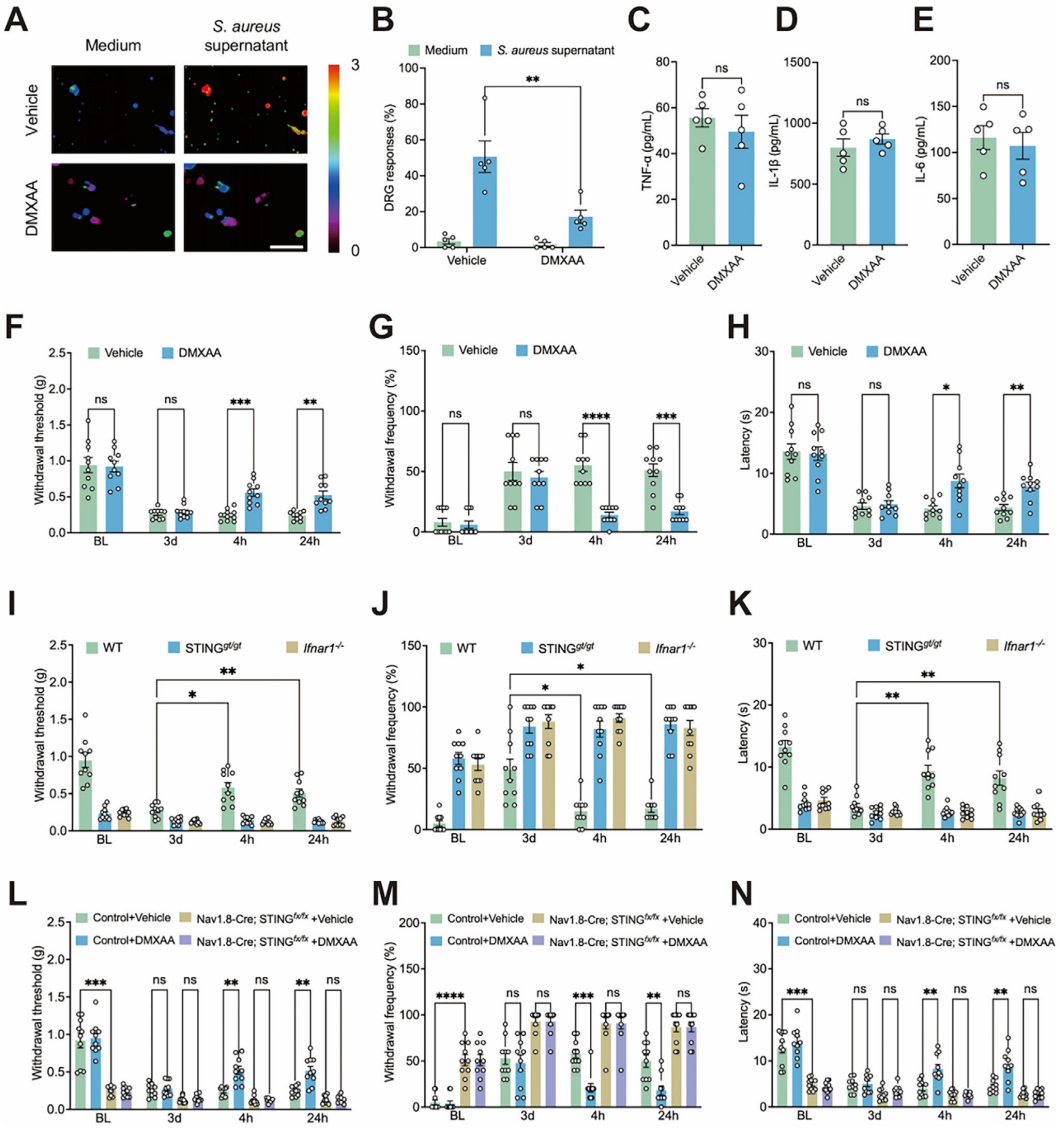
STING agonist mediates rapid analgesia via activation of the STING/IFN‐I pathway in nociceptors. (A, B) Calcium imaging of cultured L3‐L5 DRG neurons pretreated with vehicle or DMXAA (50 µm) for 4 h and then stimulated with *S. aureus* culture supernatant, showing representative fields of view (A) and the proportions of neurons activated (B) for each treatment group. (Scale bar: 50 µm; color bar, F340/F380 ratio). n = 5 wells/group; (C–E) Bone levels of TNF‐α (C), IL‐1β (D), and IL‐6 (E) in mice with *S. aureus*‐induced osteomyelitis, measured by ELISA at 4 h after vehicle or DMXAA treatment on day 3 post‐infection. n = 5 mice/group; (F–H) Temporal changes in mechanical (F, G) and thermal (H) hyperalgesia in a murine model of *S. aureus*‐induced osteomyelitis treated with vehicle or DMXAA. Pain sensitivity was assessed at baseline before infection (BL), on day 3 post‐infection before treatment, and at 4 and 24 h after treatment on day 3. n = 10 mice/group; I‐K) Temporal changes in mechanical (I, J) and thermal (K) hyperalgesia in vehicle‐ or DMXAA‐treated WT, *STING^gt/gt^* and *Ifnar1^−/−^* mice with *S. aureus*‐induced osteomyelitis. n = 10 mice/group; L‐N) Temporal changes in mechanical (L, M) and thermal (N) hyperalgesia in vehicle‐ or DMXAA‐treated control and *Nav1.8*‐*Cre*; *STING^fx/fx^* mice with *S. aureus*‐induced osteomyelitis. n = 10 mice/group; BL: Baseline. WT: Wild‐type. Control: littermate control mice (*Nav1.8*‐*Cre* ^−/−^; *STING^fx/fx^*). *Nav1.8*‐*Cre*; *STING^fx/fx^* (*Nav1.8*‐*Cre* ^+/−^; *STING^fx/fx^*). Statistical analysis: (B, F‐N) Two‐way ANOVA, Bonferroni's post‐hoc tests. (C–E) Student's *t*‐tests. ^*^ *P*<0.05, ^**^ *P*<0.01, ^***^ *P*<0.001, ^****^ *P*<0.0001. ns = not significant. All data are presented as mean ± SEM.

To verify that the STING agonist can alleviate osteomyelitis pain shortly after administration by inhibiting the activation of nociceptive neurons by pathogens, we administered DMXAA (20 mg/kg) via intraperitoneal injection 3 days post‐infection. We observed a significant analgesic effect 4 h after administration; however, at this time point, the inflammatory markers TNF‐α, IL‐1β, and IL‐6 did not show significant differences between groups (Figure 4C–H). Therefore, this immediate analgesic effect may primarily result from the inhibition of nociceptive neuron activation rather than from a reduction in inflammatory levels.

To confirm that STING agonists can indeed exert analgesic effects during osteomyelitis by activating the STING/IFN‐I signaling pathway in nociceptive neurons, we first utilized RTX to ablate nociceptive neurons in mice, creating nociceptive‐deficient models. After establishing the osteomyelitis model, we treated the mice with DMXAA. We observed that the RTX‐treated mice exhibited significantly reduced pain during osteomyelitis, and subsequent administration of DMXAA did not produce any additional analgesic effects (Figure S4A–C). This indicates that nociceptive neurons play a crucial role in the perception of osteomyelitis pain and in the analgesic modulation induced by STING agonists. To further elucidate the role of the STING/IFN‐I signaling pathway in regulating pain perception in nociceptive neurons, we bred mice with defects in STING expression specifically in Nav1.8^+^ nociceptive neurons (*Nav1.8‐Cre; STING^fx/fx^*) and mice with defects in *Ifnar1* gene expression (*Nav1.8‐Cre; Ifnar1^fx/fx^*). Similar to STING^gt/gt^ mice, the *Nav1.8‐Cre; STING^fx/fx^* mice exhibited hyperalgesia compared to control mice and did not show pain relief following DMXAA injection during osteomyelitis (Figure 4I–N). Likewise, the *Nav1.8‐Cre; Ifnar1^fx/fx^* mice also displayed similar hyperalgesia and a loss of the analgesic effect of STING agonists (Figure S4D–F). These findings indicate that the analgesic effects of DMXAA are dependent on the STING/IFN‐I signaling pathway within nociceptive neurons.

### 2.5 STING Agonists Inhibit Osteoclast Differentiation and Reduce Bone Destruction in Osteomyelitis via the STING/IFN‐I Pathway

We have observed the protective effects of STING agonists on bone tissue during the course of osteomyelitis, and this protective effect is dependent on the STING/IFN‐I signaling pathway. However, it remains unclear whether the protective actions of STING agonists on bone tissue are mediated through the regulation of local inflammation, bacterial load, osteoblast and osteoclast differentiation, or a combination of these factors.

Previous studies using ovariectomy‐induced osteoporosis mouse models have demonstrated that IFN‐I agonists can ameliorate bone loss by suppressing osteoclast differentiation [32]. Based on these findings, we hypothesized that during the progression of osteomyelitis, STING agonists may also inhibit osteoclast activity through activation of the IFN‐I pathway, thereby directly influencing the balance between bone formation and osteoclastogenesis. To investigate this, we established a *S. aureus* osteomyelitis model in mice and administered DMXAA via intraperitoneal injection on days 3 and 7 post‐infection. On day 14 post‐model establishment, we performed TRAP staining on femoral tissue sections to quantify osteoclast numbers. The results indicated that the number of osteoclasts was significantly reduced in the DMXAA‐treated mice (Figure 5A,B). Masson staining was used to compare the differences in new bone formation between the two groups, and the results showed that DMXAA treatment had no significant impact on osteogenesis (Figure 5C,D). Additionally, to further assess the activity of osteoclasts and osteoblasts, we measured serum levels of CTX‐I and PINP on day 14 post‐infection, with CTX‐I serving as a marker for bone resorption and PINP as a marker for bone formation. The results revealed that DMXAA significantly decreased CTX‐I levels, with no significant effect on PINP levels (Figure 5E,F). This indicates that STING agonists can reduce bone destruction during the course of osteomyelitis by decreasing osteoclast numbers and inhibiting their function. However, further experiments showed that treatment with DMXAA did not affect the number of osteoclasts or CTX‐I levels in STING^gt/gt^ mice and *Ifnar1^−/−^* mice, suggesting that the aforementioned regulatory effects are dependent on the STING/IFN‐I signaling pathway (Figure 5A–F).

**FIGURE 5:**
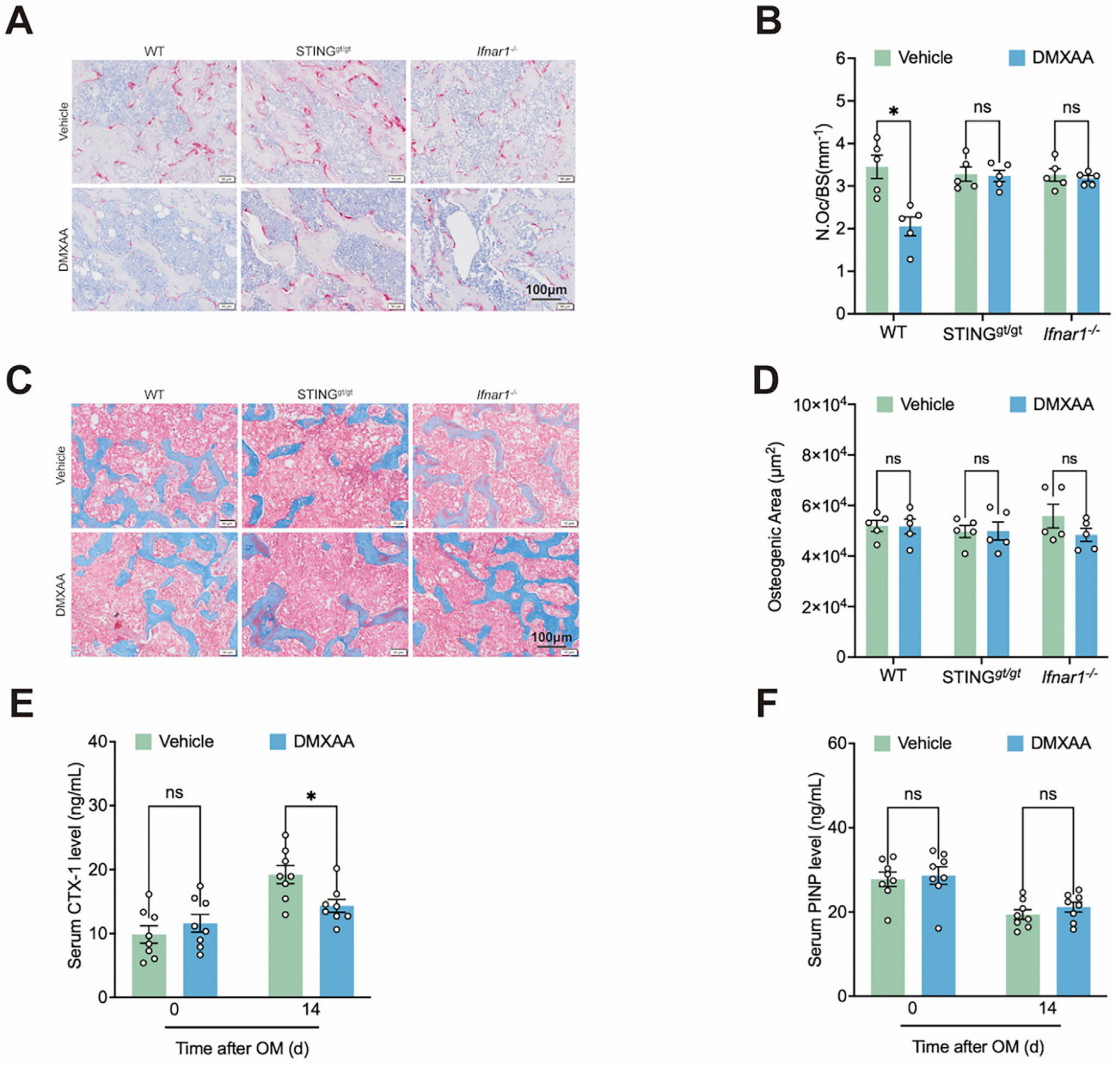
STING agonists inhibit osteoclast differentiation and reduce bone destruction in osteomyelitis via the STING/IFN‐I pathway. (A, B) Representative images (A) and quantification (B) of TRAP staining showing osteoclast numbers in the femurs of *S.aureus*‐infected WT, STING^gt/gt^, and *Ifnar1^−/−^* mice treated with vehicle or DMXAA on day 3 and day 7 post‐infection, harvested at day 14 (Scale bar: 100 µm). n = 5 mice/group; (C, D) Representative images (C) and quantification (D) of Masson's staining indicating new bone formation in the femurs of *S. aureus*‐infected WT, STING^gt/gt^, and *Ifnar1^−/−^* mice treated with vehicle or DMXAA on day 3 and day 7 post‐infection, harvested at day 14 (Scale bar: 100 µm). n = 5 mice/group; (E, F) Serum levels of CTX‐I (E) and PINP (F) measured by ELISA at day 0 (baseline) and day 14 in vehicle‐ or DMXAA‐treated mice. n = 8 mice/group. WT: Wild‐type. OM: Osteomyelitis. Statistical analysis: (B, D, E, F) Two‐way ANOVA, Bonferroni's post‐hoc tests. ^*^ *P*<0.05. ns = not significant. All data are presented as mean ± SEM.

In further validation experiments conducted in vitro, we induced the differentiation of mouse RAW 264.7 cells into osteoclasts using RANKL (50 ng/mL) for 6 days, while intervening with various doses of DMXAA (1, 10, 30, and 50 µm). The results demonstrated that DMXAA could dose‐dependently inhibit osteoclast differentiation (Figure S5A–C). To verify the role of the STING/IFN‐I signaling pathway in this process, we generated bone marrow‐derived macrophages (BMDM) from STING^gt/gt^ and *Ifnar1* ^−/−^ mice by culturing with M‐CSF (30 ng/mL) for 3 days; cells were then induced to form osteoclasts with M‐CSF (30 ng/mL) and RANKL (50 ng/mL) for 7 days in the presence or absence of 30 µm DMXAA. TRAP staining results indicated that DMXAA intervention significantly inhibited the differentiation of BMDM into osteoclasts; however, there was no notable inhibitory effect observed in BMDM derived from STING^gt/gt^ and *Ifnar1* ^−/−^ mice (Figure S5D,E). In summary, STING agonists can exert a significant inhibitory effect on osteoclast formation both in vivo and in vitro, and this effect is dependent on the STING/IFN‐I signaling pathway.

### 2.6 STING Agonist Alleviates Pain and Reduces Abscess Formation by Suppressing Inflammatory Signaling in Chronic Osteomyelitis

Previous literature has indicated that activation of the STING/IFN‐I signaling pathway can affect inflammatory responses [33], which may subsequently influence pain levels [34]. Although we have previously demonstrated that the STING agonist exerts analgesic effects by directly acting on nociceptive neurons independent of changes in inflammatory factors during the early stages of osteomyelitis (Figure 4), it remains unknown whether the analgesic effects of the STING agonist during the chronic phase of osteomyelitis depend on its suppression of inflammatory responses. To evaluate the impact of the STING agonist on inflammation level during the chronic phase of osteomyelitis, we administered equal volumes of vehicle or DMXAA (20 mg/kg) via intraperitoneal injection on days 3 and 7 post‐infection and measured the levels of TNF‐α, IL‐1β, and IL‐6 in bone tissue on days 3, 7, and 14 post‐infection. The results showed that the STING agonist DMXAA reduced the levels of IL‐1β in bone tissue, but had no significant effect on TNF‐α and IL‐6 (Figure 6A–C). To further validate the impact of inflammatory factors on pain, we constructed *S. aureus* osteomyelitis mice and treated with IL‐1β neutralizing antibody, which significantly alleviated pain in the osteomyelitis mice. Additionally, when recombinant IL‐1β was applied to the DMXAA‐treated mice, their pain levels significantly increased (Figure 6D–F). These experiments indicate that during the chronic phase of osteomyelitis, the STING agonist not only provides analgesic effects by directly targeting nociceptive neurons but also alleviates pain by reducing levels of inflammatory factors represented by IL‐1β. To determine the role of the STING/IFN‐I signaling pathway in regulating inflammation levels, we measured the levels of TNF‐α, IL‐1β, and IL‐6 in the bone tissue of STING/IFN‐I signaling‐deficient mice on day 7 post‐infection. We found that the levels of IL‐1β in the femoral tissue of STING*^gt/gt^* and *Ifnar1^−/−^* osteomyelitis mice were significantly higher than those in WT mice, while there were no significant differences in the levels of TNF‐α and IL‐6 among the three groups (Figure S6A–C).

**FIGURE 6:**
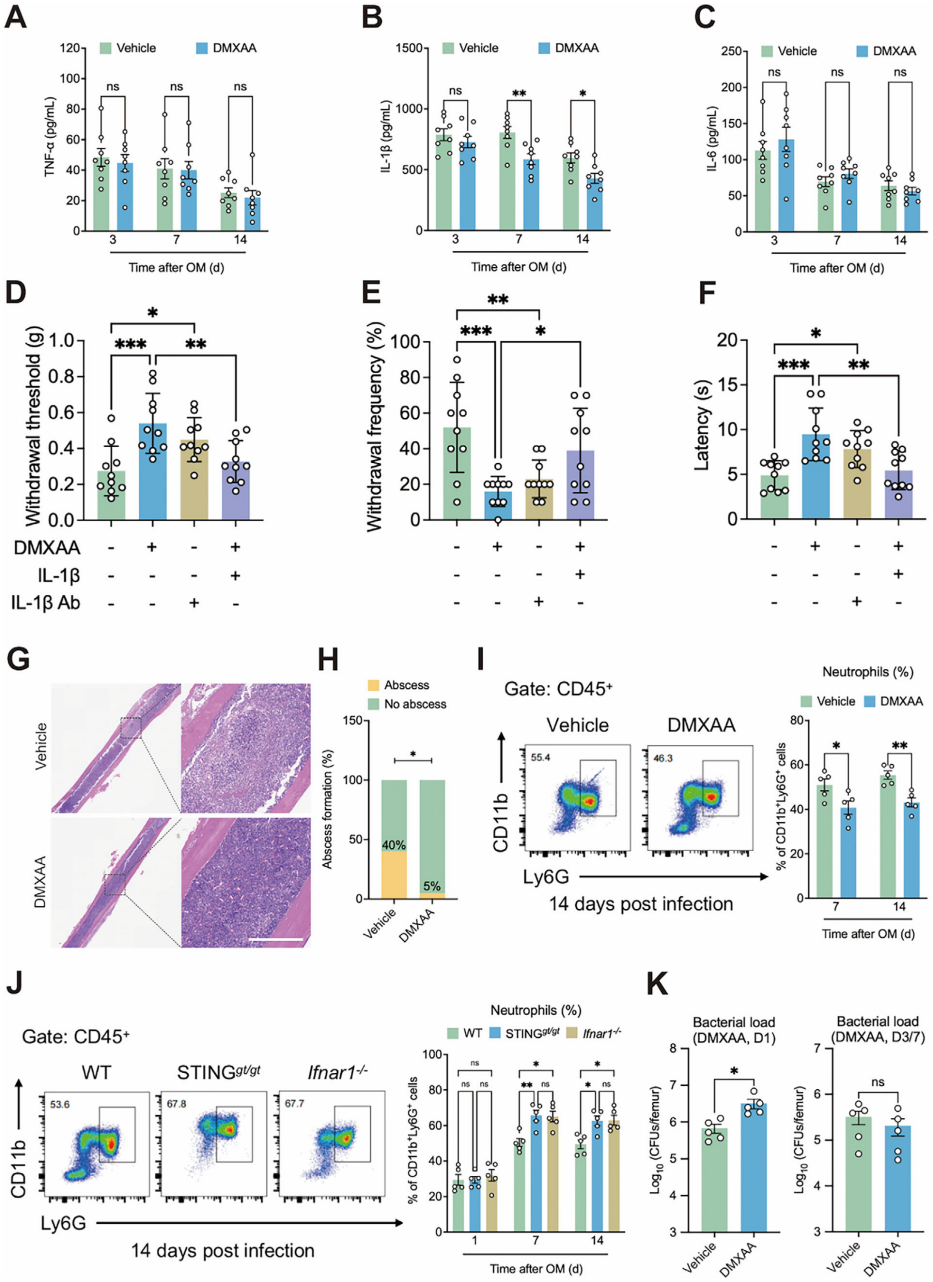
STING agonist alleviates pain and reduces abscess formation by suppressing inflammatory signaling in chronic osteomyelitis. (A–C) ELISA analysis of TNF‐α (A), IL‐1β (B), and IL‐6 (C) levels in bone tissue of mice with *S.aureus*‐induced osteomyelitis. Mice received vehicle or DMXAA (20 mg/kg, i.p.) on day 3 and day 7 post‐infection. Bone tissue was harvested at the indicated time points (days 3, 7, and 14) post‐infection. n = 8 mice/group; (D–F) Effects of IL‐1β neutralizing antibody or recombinant IL‐1β on pain sensitivity in mice with *S. aureus*‐induced osteomyelitis at day 3 post‐infection. Mechanical hyperalgesia (D, E) and thermal hyperalgesia (F) were assessed in mice treated with vehicle, DMXAA, IL‐1β neutralizing antibody (IL‐1β Ab), or DMXAA + recombinant IL‐1β. n = 10 mice/group; G, H) Representative H&E‐stained of femurs (G) and quantification of the abscess formation rate (H) in vehicle‐ or DMXAA‐treated mice with *S. aureus*‐induced osteomyelitis at day 14 post‐infection (Scale bar: 500 µm). n = 20 mice/group; I) Representative flow cytometry images on day 14 post‐infection for the Vehicle and DMXAA treatment groups (left) with quantification of neutrophil proportions (CD45^+^CD11b^+^Ly6G^+^) on days 7 and 14 (right). n = 5 mice/group; (J) Representative flow cytometry images on day 14 post‐infection for wild‐type (WT), STING^gt/gt^, and *Ifnar1^−/−^* mice (left) with quantification of neutrophil proportions on days 1, 7, and 14 (right). n = 5 mice/group; (K) Bacterial load in the femurs assessed 3 days after the last DMXAA (20 mg/kg) administration: following treatment on day 1 (left) or on days 3 and 7 (right). n = 5 mice/group. OM: Osteomyelitis. Statistical analysis: (A–C, I, J) Two‐way ANOVA, Bonferroni's post‐hoc tests. (D–F) One‐way ANOVA, Tukey's post‐hoc tests. (H) Fisher's exact tests. (K) Student's *t*‐tests. ^*^ *P*<0.05, ^**^ *P*<0.01, ^***^ *P*<0.001, ^****^ *P*<0.0001. ns = not significant. All data are presented as mean ± SEM.

Previous studies have indicated that IL‐1 can promote abscess formation [35]. We hypothesized that the STING agonist affects the formation of local abscesses in osteomyelitis by reducing the expression levels of IL‐1β. To investigate the impact of the STING agonist on abscess formation in osteomyelitis, we performed histological analysis and HE staining on the infected femurs of mice on day 14 post‐osteomyelitis modeling. The results demonstrated that after 2 weeks of osteomyelitis, the proportion of local abscess formation in the STING agonist‐treated group was significantly lower than that in the vehicle group (Figure 6G,H).

Neutrophils play a central role in the formation of abscesses associated with osteomyelitis. We analyzed the number of neutrophils in the bone marrow of infected mouse femurs at various time points post‐*S. aureus* infection using flow cytometry. After administering DMXAA on days 3 and 7 post‐infection, we observed that the proportion of neutrophils in the DMXAA group was significantly lower than that in the control group on days 7 and 14 (Figure 6I). To further elucidate the role of the STING/IFN‐I pathway in modulating immunity during the acute and chronic phases of infection, we established osteomyelitis models in STING*^gt/gt^* and *Ifnar1^−/−^* mice and subsequently conducted flow cytometric analyses of bone marrow cells. The results showed that there were no significant differences in neutrophil proportions between STING*^gt/gt^*, *Ifnar1^−/−^*, and wild‐type (WT) mice on day 1 post‐infection. However, on days 7 and 14 post‐infection, the proportion of neutrophils in STING*^gt/gt^* and *Ifnar1^−/−^* mice was higher compared to that in WT mice (Figure 6J). In summary, the STING/IFN‐I pathway significantly influences neutrophil responses during *S. aureus* osteomyelitis, inhibiting both neutrophil recruitment and subsequent abscess formation.

Abscesses are a localized response of the body to infection, and their formation helps to confine the infection to a specific area, preventing its spread to surrounding tissues or systemically. DMXAA has been shown to reduce abscess formation within the femur in cases of osteomyelitis. This raises the question of whether this reduction in abscess formation could impair the body's ability to contain pathogens. To address this concern, we conducted a bacterial load experiment in the femurs. The results indicated that administering DMXAA on the first day post‐infection increased the bacterial load in the DMXAA group. However, when administered on days 3 and 7 post‐infection, there were no significant differences in bacterial load between the DMXAA and vehicle groups (Figure 6K). Therefore, the administration of DMXAA on days 3 and 7 did not impact the ability to eliminate pathogens, but it did influence the formation of abscesses (Figure 6G). Furthermore, to evaluate whether DMXAA treatment resulted in systemic bacterial dissemination, we quantified bacterial load in the blood, livers, spleens, and kidneys of vehicle‐ and DMXAA‐treated mice at days 3, 7, and 14 post‐infection. No detectable bacteria were observed in these peripheral tissues in either group, indicating that the infection remained localized to the femur without systemic dissemination (Figure S6E–H).

### 2.7 The Abscess Inhibition Effect of STING Agonists Enhances Antibiotic Efficacy

Abscesses can impede antibiotic penetration, making it difficult for antibiotics to reach the sites of infection and effectively eradicate the bacteria [36]. Given that DMXAA can inhibit abscess formation, we hypothesized that the combination of DMXAA with antibiotics could enhance the penetration of antibiotics into the infected tissue, particularly within abscesses, thereby increasing their efficacy. We administered DMXAA to mice on days 3 and 7 post‐osteomyelitis modeling, and began daily administration of the sensitive antibiotic (vancomycin) starting on day 3. To assess the infection status within the femurs of the osteomyelitis mice, we measured the bacterial load in the femurs. The results indicated that from day 7 onward, the bacterial load in the DMXAA + antibiotic group was significantly lower than that in the Vehicle + antibiotic group (Figure 7A). To further determine whether DMXAA treatment affects the local concentration of vancomycin, vancomycin concentrations in the femurs of mice from both groups were measured on day 14 post‐infection. The results showed that DMXAA treatment significantly increased the vancomycin concentration in the femurs, suggesting that the enhanced therapeutic efficacy of DMXAA in combination with antibiotics may be associated with increased local antibiotic exposure at the site of infection (Figure S7A).

**FIGURE 7:**
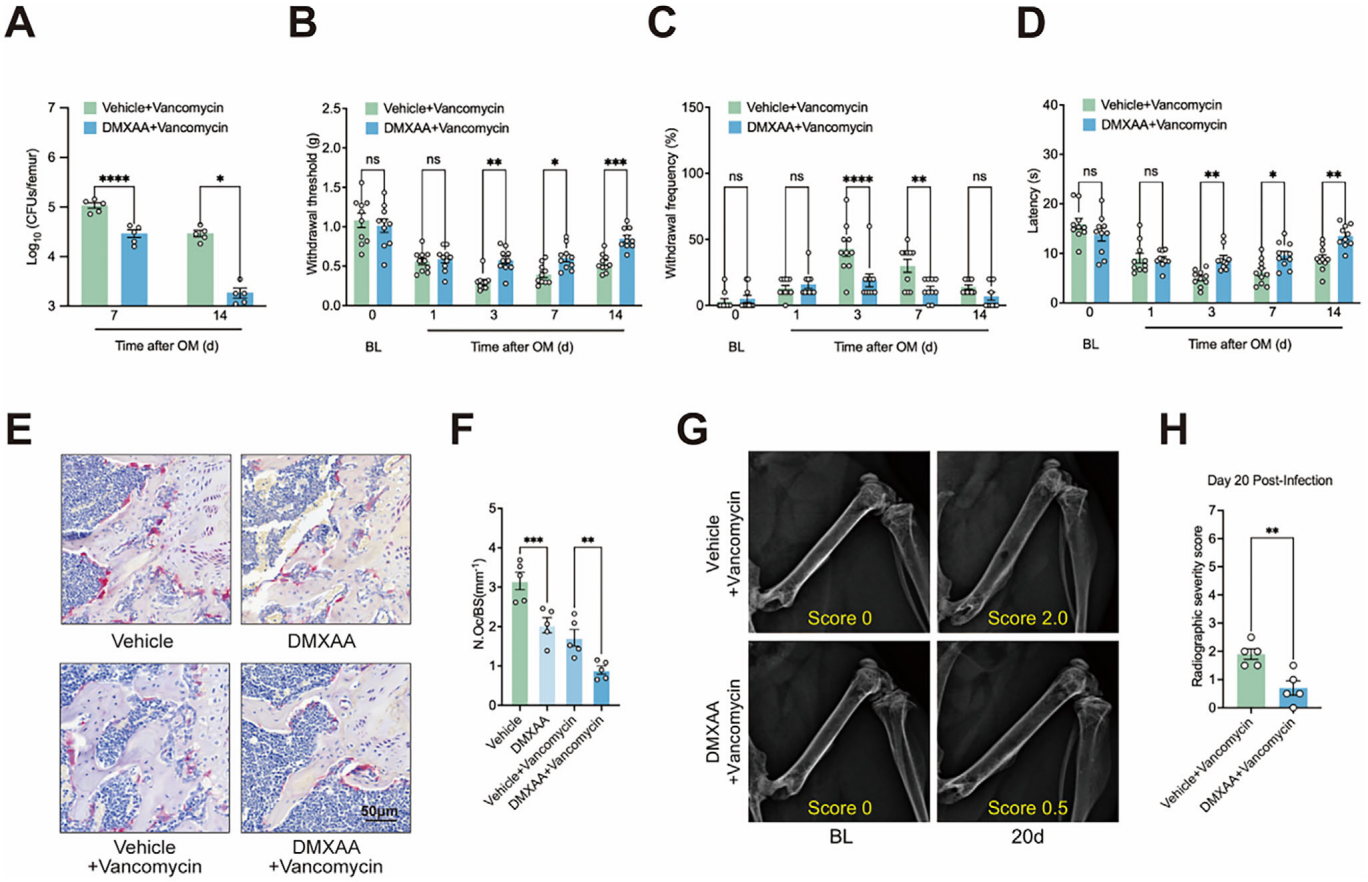
STING agonist enhances antibiotic efficacy and reduces bacterial load. (A) Bacterial load (CFU) in femurs of mice with *S. aureus*‐induced osteomyelitis at day 7 and day 14 post‐infection, treated with Vehicle + Vancomycin or DMXAA + Vancomycin. n = 5 mice/group; (B–D) Temporal changes in mechanical (B, C) and thermal (D) hyperalgesia in mice with *S. aureus*‐induced osteomyelitis treated with Vehicle+Vancomycin or DMXAA+Vancomycin. Pain sensitivity was assessed at day 0 (baseline) and days 1, 3, 7, 14 post‐infection. n = 10 mice/group; (E, F) Representative images (E) and quantification (F) of TRAP staining showing osteoclast numbers in femurs of *S. aureus*‐infected mice at day 14. (Scale bar: 50 µm). n = 5 mice/group; (G, H) Representative x‐ray images (G) and radiographic severity scores (H) of femurs at day 0 (baseline) and day 20 post‐infection in mice with *S. aureus*‐induced osteomyelitis treated with Vehicle+Vancomycin or DMXAA+Vancomycin. n = 5 mice/group. Mice with *S. aureus*‐induced osteomyelitis were treated with DMXAA (or vehicle) on day 3 and day 7 post‐infection; vancomycin was administered daily starting from day 3. OM: Osteomyelitis. BL: Baseline. Statistical analysis: (A‐D) Two‐way ANOVA, Bonferroni's post‐hoc tests. (F) One‐way ANOVA, Tukey's post‐hoc tests. (H) Student's *t*‐tests. ^*^ *P*<0.05, ^**^ *P*<0.01, ^***^ *P*<0.001, ^****^ *P*<0.0001. ns = not significant. All data are presented as mean ± SEM.

To assess pain level differences between the two mouse groups during treatment, we conducted Von Frey and Hargreaves tests for mechanical and thermal pain sensitivity, respectively. We observed that starting from day 3 post‐infection, the pain threshold in the DMXAA + antibiotic group was higher than that in the Vehicle + antibiotic group (Figure 7B–D). To clarify the impact of the combined treatment of DMXAA and antibiotics on bone destruction in the osteomyelitis mice, we conducted TRAP staining on the osteomyelitis bone tissue. The results showed that the DMXAA + antibiotic group significantly reduced the number of osteoclasts compared to the Vehicle + antibiotic group (Figure 7E,F). X‐ray imaging similarly indicated that the DMXAA + antibiotic group exhibited less bone destruction (Figure 7G,H). Furthermore, we repeated the above experiments using STING^gt/gt^ and *Ifnar1^−/−^* mice. DMXAA did not improve the therapeutic effect of the antibiotic, showing no significant results in reducing bacterial load, pain relief, or inhibition of bone destruction (Figure S7B–Q). Collectively, these results indicate that the combination of DMXAA with the sensitive antibiotic reduces bacterial load in osteomyelitis, alleviates pain, and inhibits associated bone destruction.

## 3 Discussion

In this study, we administered STING agonists to mice with methicillin‐resistant Staphylococcus aureus (MRSA)‐induced osteomyelitis. Our results demonstrate that STING activation significantly alleviated pain symptoms, suppressed infection‐mediated bone destruction, and enhanced the bactericidal efficacy of antibiotics. These multimodal therapeutic effects​ indicate that STING pathway activation exerts pleiotropic regulatory actions​ on the pathogenic processes of osteomyelitis.

Pain is one of the primary clinical manifestations of osteomyelitis and a major reason for patients seeking medical attention [3]. Currently, commonly used analgesics include nonsteroidal anti‐inflammatory drugs (NSAIDs) and opioids, both of which present unavoidable side effects. As is well known, NSAIDs can lead to gastrointestinal or cardiovascular adverse events and may inhibit osteogenic repair, raising significant concerns in diseases characterized by bone tissue destruction [37, 38]. Additionally, the addictive nature of opioids has resulted in serious societal issues [39]. This study demonstrates that STING agonists not only exhibit analgesic effects in the context of fractures and bone tumors but also provide pain relief in cases of orthopedic infections [22]. This finding offers valuable insights for the future application of STING agonists in orthopedic diseases. On the other hand, compared to bone metastases, where pain symptoms typically manifest in the later stages of the disease [40], osteomyelitis can induce pain early in its course. Thus, STING agonists could be applied earlier in the treatment of osteomyelitis, alleviating patient pain, reducing bone destruction caused by pathogens, and enhancing the effectiveness of antibiotic therapy. From this perspective, the application of STING agonists in bone and joint infections may hold even greater potential.

Consistent with previous research findings [41], this study observed a significant reduction in pain thresholds in STING knockout mice, with similar effects noted in IFNAR1 knockout mice. This indicates that the STING pathway can independently influence pain perception and plays a critical role in maintaining normal pain thresholds. STING agonists were found to alleviate pain in mice with osteomyelitis; however, this effect was lost in STING knockout and IFNAR1 knockout mice, further confirming that the action of STING agonists relies on the STING/IFN‐I signaling axis. Moreover, the analgesic mechanism of STING agonists during the course of osteomyelitis is not solely limited to affecting nociceptive neuron function; it may also involve the inhibition of osteoclast activation and the suppression of inflammatory responses [42, 43, 44].

Multiple bacterial virulence factors have been shown to activate osteoclast function [15, 45], while the inflammatory milieu during pathogenic infection further potentiates osteoclast activation [46]. These dual mechanisms collectively drive infection‐related bone destruction and pain. In this study, radiographic analysis of STING agonist‐treated groups revealed significant mitigation of bone remodeling imbalance. Notably, such improvement predominantly manifested as suppressed osteoclast‐mediated bone resorption—with no statistical differences in osteogenesis markers—indicating that STING signaling confers protection by inhibiting osteoclast differentiation or attenuating osteolytic activity, consistent with prior evidence [44]. Furthermore, this study demonstrated that STING agonists significantly reduced serum CTX‐I levels and osteoclasts in femurs in vivo. In vitro, they exerted dose‐dependent suppression of RANKL‐induced osteoclast differentiation in a STING/IFNAR1‐dependent manner. Collectively, these results establish that STING agonists directly inhibit osteoclast differentiation through the STING/IFN‐I axis, providing intrinsic bone protection independent of immunomodulatory mechanisms.

Antibiotics are a crucial treatment modality for osteomyelitis; however, various factors can diminish their bactericidal efficacy, among which abscess formation is a significant contributor [11]. Following bacterial invasion of bone tissue, neutrophil recruitment forms abscesses encased by dense fibrous tissue and necrotic bone fragments. This creates a physical barrier that severely impedes antibiotic penetration and function [47, 48, 49, 50]. Existing evidence indicates that IFN‐I activates the transcription factor STAT1 via the IFN‐I/IFNAR axis, suppressing NLRP1 and NLRP3 inflammasome activity [33, 51, 52], and subsequently reducing the transcription of IL‐1β — an important inflammatory factor in abscess formation [35]. This suggests that STING agonists may inhibit IL‐1β‐mediated abscess formation through STING/IFN‐I signaling. Our study found that at day 14 post‐infection, STING agonist‐treated mice exhibited almost no abscess formation (vs. 40% incidence in controls). Moreover, combining STING agonists with antibiotics achieved significantly lower bacterial load on days 7 to 14 compared to antibiotic monotherapy. This effect was not observed in STING^gt/gt^ and Ifnar1^−/−^ mice, demonstrating that the STING agonist relies on the STING/IFN‐I signaling pathway to inhibit abscess formation and enhance the bactericidal effect of antibiotics.

## 4 Conclusion

The STING/IFN‐I signaling pathway plays an important role in pain pathogenesis and bone destruction during osteomyelitis. Specifically: (1) STING agonists directly target nociceptors to alleviate osteomyelitic pain via STING/IFN‐I axis activation; (2) pathway activation suppresses osteoclast differentiation, thereby mitigating pathogen‐induced bone destruction; (3) by reducing IL‐1β production, STING activation potently inhibits abscess formation, thereby augmenting the bactericidal efficacy of antibiotics.

## 5 Methods

### 5.1 Animals

All animal care and experiments were approved by our animal protection and use committee (IACUC FJMU 2022‐NSFC‐0368). The animals were kept in individually ventilated miniature isolation cages with free access to water and food at a temperature of 22°C–24°C, a humidity of 60% ± 5%, and a 12 h light/dark cycle. C57BL/6J mice were purchased from Beijing HFK Bioscience Co., Ltd. *Nav1.8‐Cre* mice were provided by Professor Qiufu Ma (Harvard Medical School, Boston) and Professor Longzhen Cheng (Southern University of Science and Technology, Shenzhen). STING*^gt/gt^* (C57BL/6J‐*Sting1^gt^*/J, JAX 017537) mice were purchased from Jackson Laboratory. *Ifnar1^−/−^* (C57BL/6Smoc‐Ifnar1^em1Smoc^, NM‐KO‐18038), STING*^fx/fx^* (B6;129S‐Tmem173^tm1(flox)Smoc^, NM‐CKO‐00014) and *Ifnar1^fx/fx^* (C57BL/6Smoc‐Ifnar1^em1(flox)Smoc^, NM‐CKO‐200100) mice were purchased from Shanghai Model Organisms Center, Inc. C57BL/6Smoc‐Ifnar1^em1Smoc^ (NM‐KO‐18038) heterozygous mice were bred together to produce both IFNAR1‐knockout (*Ifnar1^−/−^*) mice and littermate control (*Ifnar1^+/−^*) mice. For the conditional knockout experiments, we generated STING*^fx/fx^* (B6;129S‐Tmem173^tm1(flox)Smoc^, NM‐CKO‐00014) and *Ifnar1^fx/fx^* (C57BL/6Smoc‐Ifnar1^em1(flox)Smoc^, NM‐CKO‐200100) mice. These mice were then bred with *Nav1.8‐Cre^+/−^* mice to generate mice with specific depletion of STING or IFNAR1 in Nav1.8^+^ neurons (*Nav1.8*‐*Cre* ^+/−^; STING*^fx/fx^* or *Nav1.8*‐*Cre* ^+/−^; *Ifnar1^fx/fx^*), and their respective control littermates (*Nav1.8*‐*Cre^−/−^*; STING*^fx/fx^* or *Nav1.8*‐*Cre^−/−^*; *Ifnar1^fx/fx^*). For all experiments conducted in this study, both male and female mice ranging from 8–14 weeks of age were used, with age matching ensured between groups.

### 5.2 Bacterial Strains and Cultures

The MRSA strain USA300 and *Streptococcus pyogenes* ATCC19615 were used in this study. The bacterial stocks were stored at −80°C in glycerol. The stocks were recovered on LB agar (Sangon Biotech) at 37°C and 5% CO_2_ for 18–22 h before use. Bacterial suspensions were prepared in sterile saline and quantified by measuring the optical density.

### 5.3 Construction of the Femoral Osteomyelitis Mouse Model

Mice were anesthetized via inhalation of 3% isoflurane, and the right hind limb was shaved to ensure a clean surgical site. The limb was then disinfected with 75% ethanol. A surgical incision, approximately 3 mm in length, was made along the dorsal aspect of the thigh in alignment with the femur using a scalpel. Careful dissection with forceps was employed to expose the dorsal surface of the femur. A micro drill was utilized to create a hole on the dorsal side of the femur. A Hamilton micro‐syringe fitted with a 34G needle was used to inject 2 µL (1 × 10^6^ CFU) of bacterial suspension or sterile saline into each femur. Following the injection, the needle was retained in position for 5 s before being withdrawn slowly to minimize trauma. Bone wax was applied to seal the drilled hole, thereby preventing bleeding and leakage of the bacterial suspension. The surgical incision was closed with sterile sutures, and the area was disinfected once more with 75% ethanol. All procedures were conducted under stringent aseptic conditions to mitigate the risk of contamination. The successful establishment of the osteomyelitis model in individual animals was defined based on the presence of characteristic clinical signs, including redness and swelling of the affected limb, accompanied by decreased pain thresholds.

### 5.4 Drug Administration

DMXAA was dissolved in sterile PBS containing 0.75% NaHCO_3_ to a target concentration of 20 mg/mL. Similarly, ADU‐S100 was prepared in sterile PBS at a concentration of 20 mg/mL. Both drugs were administered via intraperitoneal injection at a dose of 20 mg/kg on days 3 and 7 following the osteomyelitis model induction. To investigate the effect of IL‐1β on pain associated with osteomyelitis, we performed daily intraperitoneal injections of either anti–IL‐1β antibody (2 mg/kg, Thermo Fisher Scientific) or IL‐1β (400 ng/kg, MedChemExpress) according to the experimental design, and pain sensitivity was assessed 4 h after the intraperitoneal injection of DMXAA (20 mg/kg) on the third day post‐infection. Additionally, to evaluate the combination effect of DMXAA with antibiotics, vancomycin (50 mg/kg, MedChemExpress) was administered daily via intraperitoneal injection starting from day 3 after the induction of osteomyelitis.

### 5.5 Behavior Tests

Mechanical hyperalgesia was evaluated using Von Frey filaments and the up‐down method [53]. The 50% paw withdrawal threshold was calculated using the formula: 10 ^(Xf+kδ)/10,000^, where Xf represents the value of the final Von Frey filament (in logarithmic units), k is derived from the response pattern, and δ is the mean difference between stimuli (also in logarithmic units) [54]. Additionally, paw withdrawal frequency was measured to reflect changes in mechanical hyperalgesia. Specifically, a 0.16 g Von Frey filament was used to repeatedly stimulate the paw 10 times, with a 1 min interval between each stimulation. The number of withdrawals recorded during these 10 stimuli was used to calculate paw withdrawal frequency as follows: paw withdrawal frequency = number of withdrawals /10% ×100%. Thermal hyperalgesia was assessed using a Hargreaves apparatus (Ugo Basile, Italy) [55]. Mice were placed on the glass surface of the device and allowed to acclimate until exploratory behavior ceased. Radiant heat was applied to the plantar surface. Paw withdrawal latency was defined as the time taken for the mouse to withdraw its paw from the thermal stimulus. A cutoff time of 30 s was implemented to prevent potential tissue damage. The rotarod test was conducted to evaluate motor coordination in mice following osteomyelitis modeling. All mice were acclimated to the rotarod by being placed on the apparatus and rotated at 4 rpm for 10 min each day for three days prior to the experimental session. During the formal experiment, mice were placed on an accelerating rotarod with an initial rotation speed set at 4 rpm, which gradually increased at a rate of 0.1 rpm/s. The time taken for each mouse to fall was recorded. The open field test was utilized to assess the exploratory activity of mice. Each mouse was placed in a 40 × 40 cm chamber, where they were monitored for 15 min using a camera positioned directly above the arena. The distance traveled by the mice was analyzed using ANY‐Maze software.

### 5.6 X‐ray Examination and Radiological Scoring

X‐ray assessments of the right femur in a prone position were conducted on mice prior to the induction of femoral osteomyelitis and at 10‐ and 20‐days post‐surgery using a KUBTEC animal x‐ray system. These imaging evaluations were performed to observe the degree of bone destruction in the femur. The osteomyelitis radiographic scoring system, adapted from Norden [29], evaluates four variables (total score 0–7; higher scores indicate worse pathology): (1) Sequestrum formation: present (1 point), absent (0); (2) Periosteal new bone formation: present (1 point), equivocal (0.5), absent (0); (3) Bone destruction severity: severe with multiple areas (2 points), moderate in one area (1), mild in one area (0.5), none (0); (4) Disease extent: proximal/mid/distal femur scored independently per region as present (1), equivocal (0.5), absent (0) (max 3 points). Total score = (Sequestrum + Periosteal + Destruction scores) + (Proximal + Mid + Distal scores).

### 5.7 Micro‐CT

The femurs of mice from different groups were collected and fixed in 4% paraformaldehyde at room temperature for one day. Following fixation, the femurs were washed three times with PBS and subsequently stored in 70% ethanol for micro‐CT analysis. Micro‐CT imaging was conducted using a Skyscan 1172 scanner (Bruker, Kontich, Belgium). Images from each group were reconstructed using consistent thresholds to facilitate 3D structural rendering of each femur. The quantified parameters included bone volume/total volume (BV/TV), trabecular number (Tb.N), trabecular thickness (Tb.Th), and trabecular separation (Tb.Sp). The extent of cortical bone destruction was calculated by subtracting the cortical bone value measured for each femur in the respective experimental groups from the mean cortical bone value of the non‐infected, sterile saline‐inoculated group.

### 5.8 ELISA

For blood samples, whole blood collected in serum separator tubes was allowed to stand at room temperature for 2 h. Subsequently, the samples were centrifuged at 1000 × g for 20 min to obtain the serum from the supernatant. For bone tissue samples, tissues were frozen in liquid nitrogen, thoroughly ground, and lysed in RIPA tissue lysis buffer (Yoche Biotech) containing 1% proteinase inhibitor and 1% phosphatase inhibitor (Yoche Biotech) on ice for 30 min. The lysates were then centrifuged at 4°C, and the supernatants were collected. Mouse ALT, AST, Cr, BUN, CTX‐1, and PINP ELISA kits were obtained from Elabscience, while mouse IL‐1β, IL‐6, TNF‐α, IFN‐α, and IFN‐β ELISA kits were sourced from R&D Systems. The Vancomycin ELISA Kit was purchased from Biorbyt. The ELISA assays were performed according to the manufacturer's instructions.

### 5.9 DRG Neuron Culture and Calcium Imaging

DRG neurons were isolated and cultured as described previously [56]. After euthanizing, DRGs were obtained from 8‐week‐old mice under sterile conditions. The intact vertebral columns were excised, and the lumbar vertebrae (L3‐L5) were separated using sterilized microscissors and microforceps under a dissection microscope. The vertebral canals were opened along the midline with microscissors, and residual spinal cord tissue was meticulously removed. L3‐L5 DRGs were then carefully dissected through the intervertebral foramen. The harvested DRGs were placed in Neurobasal A medium (Thermo Fisher) and finely minced with microscissors. The minced tissue was centrifuged at 1000 rpm for 3 min, and the supernatant was discarded. The minced tissue was then digested in 2 mL of an enzymatic solution containing collagenase A (1.25 mg/mL, Sigma‒Aldrich) and dispase II (2.5 mg/mL, Sigma‒Aldrich) while shaking in an incubator at 37°C and 150 rpm for 15 min. Following centrifugation at 1000 rpm for 5 min at 4°C, the supernatant was removed. The remaining tissue was resuspended in 1 mL of Neurobasal A medium supplemented with 10% FBS to terminate digestion, and then incubated with 150 U/mL DNAse I (TransGen Biotech). To achieve a single‐cell suspension, mechanical trituration was performed by sequentially aspirating and ejecting the DRG mixture through sterile needles of decreasing diameters (18, 21, and 26G). The cell suspension was then filtered using a 70 µm cell strainer and centrifuged at 1000 rpm for 5 min at 4°C. Finally, the dissociated DRG cells were resuspended in DRG culture medium, consisting of Neurobasal A medium supplemented with 50 ng/mL nerve growth factor (NGF, Thermo Fisher) and 10 µm cytosine arabinoside (Sigma), and then plating on plates coated with 10 µg/mL laminin.

DRG neurons were cultured on 35 mm glass‐bottom dishes for one day prior to the experiment. The neurons were loaded with 5 µm Fura‐2AM (Thermo Fisher) at 37°C for 30 min. Following a wash with PBS to remove excess dye, they were incubated in Ringer's solution. Intracellular calcium levels were monitored using an IX83 inverted fluorescence microscope (Olympus) equipped with an opti‐MOS^TM^ sCMOS camera (QImaging). During imaging, bacterial supernatants from *S. aureus* (1 × 10^9^ CFU), culture medium, vehicle, or DMXAA (APExBIO, 30 µm) were delivered to the neurons via a Multichannel Systems PPS2 perfusion system (Harvard Bioscience). Fura‐2AM was excited using alternating ultraviolet light at wavelengths of 340 and 380 nm, allowing for the detection of both calcium‐bound and free forms. Image acquisition and analysis were performed using CellSens software. The relative changes in intracellular calcium levels were represented by the ratio of emission intensity following excitation at 340 nm to that at 380 nm (F340/F380). An increase of 10% or greater compared with baseline in F340/F380 was considered indicative of elevated intracellular calcium levels.

### 5.10 Osteoclastogenesis Induction and TRAP Staining

RAW 264.7 cells and primary mouse bone marrow cells were utilized to investigate the regulatory effects of the STING agonist on osteoclastogenesis in vitro. For RAW 264.7 cells, they were cultured in DMEM supplemented with 10% FBS and 1% penicillin‐streptomycin under conditions of 37°C with 5% CO_2_. RANKL (PeproTech) at a concentration of 50 ng/mL, along with varying doses of DMXAA (0, 1, 10, 30, or 50 µm), was added to induce osteoclastogenesis. The culture medium was replaced every two days, and observations were made after six days. For the primary mouse bone marrow cells, after euthanizing the mice, femurs were aseptically isolated, and the bone marrow cells were flushed out using a sterile syringe with PBS. Following filtration and red blood cell lysis, the cells were centrifuged, and the supernatant was discarded to obtain bone marrow cells. These cells were cultured with M‐CSF (30 ng/mL, MedChemExpress) for 3 days to generate bone marrow‐derived macrophages (BMDMs). The BMDMs were then induced to form osteoclasts using M‐CSF (30 ng/mL) and RANKL (50 ng/mL) for 7 days, with or without the addition of 30 µm DMXAA. TRAP staining was performed following the manufacturer's instructions (Servicebio, China).

### 5.11 Histology

After euthanasia, mouse hearts were perfused with 50 mL of PBS followed by 50 mL of 4% paraformaldehyde (PFA) for fixation. The extracted femurs were immersed in 4% PFA for 24 h. After a 2‐week decalcification in 0.5 m EDTA, the femurs were dehydrated in an ascending ethanol gradient (70%–100%) before being embedded in paraffin. Sections of 5 µm thickness were then prepared in a sagittal orientation using a microtome (Thermo, Germany). Prior to staining, the paraffin‐embedded sections were dewaxed and rehydrated. Hematoxylin and eosin (H&E) staining, TRAP staining, and Masson staining were performed according to the manufacturer's instructions (Servicebio, China).

### 5.12 Flow Cytometry

After euthanasia, mouse femurs were harvested, thoroughly minced, and digested in 1 mL of RPMI 1640 medium containing 1.25 mg/mL collagenase A and 2.5 mg/mL dispase II, shaken at 37°C for 1 h. The resulting cell suspension was filtered through a 70 µm cell strainer to obtain a single‐cell suspension. Following a wash, the cells were incubated with FcR blockers (BD Biosciences, 553142, 1:100) on ice for 10 min, and subsequently stained with the following antibodies on ice for 30 min: anti‐mouse CD45, PE‐Cy5 (BioLegend, 103110, 1:100); anti‐mouse CD11b, BV421 (BioLegend, 101236, 1:100); anti‐mouse Ly6G, FITC (BioLegend, 127606, 1:100). After another wash, the cells were resuspended in HBSS. Flow cytometry was performed using a Beckman CytoFLEX, and the data were analyzed with FlowJo software (v10).

### 5.13 Bacterial Load Measurement

To determine bacterial dissemination and tissue bacterial load, approximately 100 µL of blood was collected from the orbital sinus of mice prior to euthanasia. After euthanasia, the femurs, livers, spleens, and kidneys were promptly harvested and transferred into sterile Eppendorf tubes. The collected tissues were homogenized in sterile saline using an automated homogenizer (Jingxin Industrial Development Co., Shanghai, China) at 60 Hz for 300 s. Blood and tissue homogenates were serially diluted and plated on LB agar (Sangon Biotech), followed by incubation overnight at 37°C with 5% CO_2_. The bacterial load was quantified by counting the number of colony‐forming units (CFUs).

### 5.14 Statistical Analysis

Statistical analyses were performed with SPSS 25.0 and GraphPad Prism 9.0 software. Unpaired Student's *t*‐test was used to compare single variables between two groups. One‐way ANOVA followed by Tukey's post–hoc test was used to compare more than two groups, and two‐way ANOVA followed by Bonferroni's was used for multiple comparisons. Fisher's exact test was employed to compare the ratio. The sample size (n) for each experiment is indicated in the corresponding figure legend. Statistical significance was established at *P* < 0.05. Values were expressed as mean ± SEM.

## Author Contributions

**Yang Chen**: conceptualization, investigation, methodology, validation, formal analysis, visualization, writing – original draft, writing – review and editing, funding acquisition. **Jianhua Lv**: conceptualization, investigation, methodology, validation, formal analysis, visualization, writing – original draft, writing – review and editing, funding acquisition. **Yuanqing Cai**: conceptualization, investigation, writing – original draft, methodology, validation, visualization, writing – review and editing, formal analysis, funding acquisition. **Xuhui Yuan**: conceptualization, methodology, validation, investigation, writing – original draft, writing – review and editing, visualization, formal analysis. **Hongxin Hu**: validation, investigation. **Jiayu Li**: investigation, validation. **Yiming Lin**: investigation. **Hongyan Li**: investigation. **Lan Lin**: investigation. **Huangfeng Lin**: investigation. **Chaofan Zhang**: investigation. **Qijin Wang**: investigation. **Ying Huang**: supervision. **Zida Huang**: supervision. **Wenbo Li**: supervision. **Susheng Niu**: resources. **Jianhua Lin**: resources. **Bin Yang**: resources, project administration, writing – review and editing, conceptualization, supervision. **Xinyu Fang**: conceptualization, writing – review and editing, project administration, resources, supervision, funding acquisition, data curation. **Wenming Zhang**: conceptualization, writing – review and editing, project administration, resources, supervision, funding acquisition, data curation.

## Conflicts of Interest

The authors declare no conflicts of interest.

## Supporting information




**Supporting File**: advs77892‐sup‐0001‐SuppMat.docx.

## Data Availability

The data that support the findings of this study are available from the corresponding author upon reasonable request.
