# Supplementary material for: STING/Type I Interferon Pathway Activation Confers Multimodal Therapeutic Benefits in Osteomyelitis
Source: Adv Sci (Weinh). 2026 Oct 26:e77892. Online ahead of print. doi: 10.1002/advs.77892 (PMC13710701; doi:10.1002/advs.77892)
Supplement: Supplementary file 1 [file ADVS-9999-e77892-s001.docx]

**Supplementary Materials for**

**STING/Type I Interferon Pathway Activation Confers Multimodal Therapeutic Benefits in Osteomyelitis**

**Supplementary Figure 1.** A-C) Temporal changes in mechanical (A, B) and thermal (C) hyperalgesia in vehicle- or DMXAA-treated mice with *Streptococcus*-induced osteomyelitis. n=10 mice/group; D-F) Temporal changes in mechanical (D, E) and thermal (F) hyperalgesia in vehicle- or ADU-S100-treated mice with *S. aureus*-induced osteomyelitis. n=10 mice/group. The time point labeled ‘0’ signiﬁes the baseline condition prior to the onset of infection. BL: Baseline. OM: Osteomyelitis. Statistical analysis: (A-F) Two-way ANOVA, Bonferroni’s post-hoc tests. *P<0.05, **P<0.01, ***P<0.001, ****P<0.0001. ns = not significant. All data are presented as mean ± SEM.

**Supplementary Figure 2.** A, B) Typical X-ray images of femur (A) and radiographic severity scores (B) assess bone destruction in *S. aureus*-induced osteomyelitis at days 10 and 20 post-infection in vehicle- or ADU-S100-treated mice. n = 8 mice/group; C-E) Comparison of motor function between vehicle- and ADU-S100-treated mice with osteomyelitis, showing latency to fall (s) in the rotarod test at baseline, day 7, and day 14 post-infection (C), and representative open field traces from a 15-minute test (D) with quantification of total distance traveled (E) at day 14 post-infection. The time point labeled ‘0’ signiﬁes the baseline condition prior to the onset of infection. n = 5 mice/group. BL: Baseline. OM: Osteomyelitis. Statistical analysis: (B, C) Two-way ANOVA, Bonferroni’s post-hoc tests. (E) Student’s t tests. *P<0.05, **P<0.01, ***P<0.001. ns = not significant. All data are presented as mean ± SEM.

**Supplementary Figure 3.** A-C) Temporal changes in mechanical (A, B) and thermal (C) hyperalgesia in vehicle- or DMXAA-treated WT and STING*^gt/gt^* mice with *S. aureus*-induced osteomyelitis. n=10 mice/group; D, E) Typical X-ray images of femur (D) and radiographic severity scores (E) assess bone destruction in *S. aureus*-induced osteomyelitis at days 10 and 20 post-infection in WT and STING*^gt/gt^* mice treated with either vehicle or DMXAA. n=8 mice/group; F) Temporal changes in body weight after osteomyelitis induction in vehicle- or DMXAA-treated WT and STING*^gt/gt^* mice. n=10 mice/group. BL: Baseline. WT: Wild-type. OM: Osteomyelitis. Statistical analysis: (A, B, C, E, F) Two-way ANOVA, Bonferroni’s post-hoc tests. *P<0.05, **P<0.01, ***P<0.001, ****P<0.0001. ns = not significant. All data are presented as mean ± SEM.

**Supplementary Figure 4.** A-C) Temporal changes in mechanical (A, B) and thermal (C) hyperalgesia in RTX-pretreated mice with *S. aureus*-induced osteomyelitis, following vehicle or DMXAA treatment on day 3 post-infection. Pain sensitivity was assessed at baseline before infection (BL), on day 3 post-infection before treatment, and at 4 and 24 hours after treatment on day 3. n=10 mice/group; D-F) Temporal changes in mechanical (D, E) and thermal (F) hyperalgesia in vehicle- or DMXAA-treated control and *Nav1.8-Cre*; *Ifnar1^fx/fx^* mice with *S.aureus*-induced osteomyelitis. n=10 mice/group. BL: Baseline. Control: littermate control mice (*Nav1.8*-*Cre*^-/-^; *Ifnar1^fx/fx^*). *Nav1.8*-*Cre*; *Ifnar1^fx/fx^* (*Nav1.8*-*Cre*^+/-^; *Ifnar1^fx/fx^*). Statistical analysis: (A-F) Two-way ANOVA, Bonferroni’s post-hoc tests. *P<0.05, **P<0.01, ***P<0.001, ****P<0.0001. ns = not significant. All data are presented as mean ± SEM.

**Supplementary Figure 5.** A-C) TRAP staining revealing osteoclast differentiation from RAW264.7 cells stimulated with 50 ng/ml RANKL for 6 days, in the presence of increasing concentrations of DMXAA (1, 10, 30, 50 µM). Representative TRAP-stained images (A), quantification of osteoclast numbers (B) and total area resorbed (C) (Scale bar: 200 µm). n = 5 wells/group; D-E) TRAP staining for osteoclasts differentiated from bone marrow-derived macrophages (BMDMs) of WT, STING^gt/gt^, or *Ifnar1^–/–^* mice. Representative TRAP-stained images (D) and quantification of osteoclast numbers (E) (Scale bar: 100 µm). n = 5 wells/group. OM: Osteomyelitis. Statistical analysis: (B, C) One-way ANOVA, Tukey’s post-hoc tests. (E) Two-way ANOVA, Bonferroni’s post-hoc tests. ***P<0.001, ****P<0.0001. ns = not significant. All data are presented as mean ± SEM.

**Supplementary Figure 6.** A-C) ELISA analysis of TNF-α (A), IL-1β (B), and IL-6 (C) levels in bone tissue of WT, STING*^gt/gt^*, and *Ifnar1^-/-^* mice with *S. aureus*-induced osteomyelitis at day 7 post-infection. n = 8 mice/group; D) Gating strategy for flow cytometry; E-H) Bacterial load (CFU) in blood (E), liver (F), spleen (G), or kidney (H) of mice with *S. aureus*-induced osteomyelitis at days 3, 7, 14 post-infection, treated with Vehicle or DMXAA. n = 5 mice/group. Statistical analysis: (A-C) One-way ANOVA, Tukey’s post-hoc tests. (E-H) Two-way ANOVA, Bonferroni’s post-hoc tests. **P<0.01. ns = not significant. All data are presented as mean ± SEM.

**Supplementary Figure 7.** A) Vancomycin concentration in femurs of mice with *S. aureus*-induced osteomyelitis at day 14 post-infection, treated with Vehicle + Vancomycin or DMXAA + Vancomycin. n = 5 mice/group. B) Bacterial load (CFU) in femurs of STING*^gt/gt^* mice with *S.aureus*-induced osteomyelitis at day 7 and day 14 post-infection, treated with Vehicle+Vancomycin or DMXAA+Vancomycin. n = 5 mice/group; C-E) Temporal changes in mechanical (C, D) and thermal (E) hyperalgesia in STING*^gt/gt^* mice with *S. aureus*-induced osteomyelitis treated with Vehicle + Vancomycin or DMXAA + Vancomycin. Pain sensitivity was assessed at day 0 (baseline) and days 1, 3, 7, 14 post-infection. n = 10 mice/group; F, G) Representative images (F) and quantification (G) of TRAP staining showing osteoclast numbers in femurs of *S. aureus*-infected STING*^gt/gt^* mice at day 14. (Scale bar: 50 µm). n = 5 mice/group; H, I) Representative X-ray images (H) and radiographic severity scores (I) of femurs at day 0 (baseline) and day 20 post-infection in STING*^gt/gt^* mice with *S. aureus*-induced osteomyelitis treated with Vehicle + Vancomycin or DMXAA + Vancomycin. n = 5 mice/group; J) Bacterial load (CFU) in femurs of *Ifnar1^-/-^* mice with *S. aureus*-induced osteomyelitis at day 7 and day 14 post-infection, treated with Vehicle+Vancomycin or DMXAA+Vancomycin. n = 5 mice/group; K-M) Temporal changes in mechanical (K, L) and thermal (M) hyperalgesia in *Ifnar1^-/-^* mice with *S. aureus*-induced osteomyelitis treated with Vehicle + Vancomycin or DMXAA + Vancomycin. Pain sensitivity was assessed at day 0 (baseline) and days 1, 3, 7, 14 post-infection. n = 10 mice/group; N, O) Representative images (N) and quantification (O) of TRAP staining showing osteoclast numbers in femurs of *S.aureus*-infected *Ifnar1^-/-^* mice at day 14. (Scale bar: 50 µm). n = 5 mice/group; P, Q) Representative X-ray images (P) and radiographic severity scores (Q) of femurs at day 0 (baseline) and day 20 post-infection in *Ifnar1^-/-^* mice with *S. aureus*-induced osteomyelitis treated with Vehicle + Vancomycin or DMXAA + Vancomycin. n = 5 mice/group. All mice with *S.aureus*-induced osteomyelitis were treated with DMXAA (or vehicle) on day 3 and day 7 post-infection; vancomycin was administered daily starting from day 3. OM: Osteomyelitis. BL: Baseline. Statistical analysis: (A, I, Q) Student’s t tests. (B-E, J-M) Two-way ANOVA, Bonferroni’s post-hoc tests. (G, O) One-way ANOVA, Tukey’s post-hoc tests. *P<0.05, **P<0.01, ***P<0.001, ****P<0.0001. ns = not significant. All data are presented as mean ± SEM.
